# Reference Values for Inward Displacement in the Normal Left Ventricle: A Novel Method of Regional Left Ventricular Function Assessment

**DOI:** 10.3390/jcdd10120474

**Published:** 2023-11-24

**Authors:** Romy R. M. J. J. Hegeman, Sean McManus, Attila Tóth, Ricardo Ladeiras-Lopes, Pieter Kitslaar, Viet Bui, Kayleigh Dukker, Serge C. Harb, Martin J. Swaans, Ori Ben-Yehuda, Patrick Klein, Rishi Puri

**Affiliations:** 1 Department of Cardiothoracic Surgery, Sint Antonius Hospital, 3435 CM Nieuwegein, The Netherlands; 2 Department of Cardiothoracic Surgery, Amsterdam University Medical Center, 1105 AZ Amsterdam, The Netherlands; 3 Bioventrix Inc., Mansfield, MA 02048, USA; 4 Department of Radiology, Gottsegen György Hungarian Institute of Cardiology & Semmelweis University, 1096 Budapest, Hungary; 5 Department of Cardiology, Gaia/Espinho Hospital Centre, Rua Conceicao Fernandes, 4434-502 Vila Nova de Gaia, Portugal; 6 Medis Medical Imaging Systems, 2316 XG Leiden, The Netherlands; 7 Department of Cardiovascular Medicine, Heart, Vascular & Thoracic Institute, Cleveland Clinic, Cleveland, OH 44195, USApurir@ccf.org (R.P.); 8 Department of Cardiology, Sint Antonius Hospital, 3435 CM Nieuwegein, The Netherlands; 9 Sulpizio Cardiovascular Center, University of California San Diego, La Jolla, CA 92037, USA

**Keywords:** inward displacement, strain, HFrEF, feature tracking, speckle tracking echocardiography

## Abstract

Background: Regional functional left ventricular (LV) assessment using current imaging techniques remains limited. Inward displacement (InD) has been developed as a novel technique to assess regional LV function via measurement of the regional displacement of the LV endocardial border across each of the 17 LV segments. Currently, normal ranges for InD are not available for clinical use. The aim of this study was to validate the normal reference limits of InD in healthy adults across all LV segments. Methods: InD was analyzed in 120 healthy subjects with a normal LV ejection fraction, using the three standard long-axis views obtained during cardiac MRI that quantified the degree of inward endocardial wall motion towards the true LV center of contraction. For all LV segments, InD was measured in mm and expressed as a percentage of the theoretical degree of maximal segment contraction towards the true LV centerline. The arithmetic average InD was obtained for each of the 17 segments. The LV was divided into three regions, obtaining average InD at the LV base (segments 1–6), mid-cavity (segments 7–12) and apex (segments 13–17). Results: Average InD was 33.4 ± 4.3%. InD was higher in basal and mid-cavity LV segments (32.8 ± 4.1% and 38.1 ± 5.8%) compared to apical LV segments (28.6 ± 7.7%). Interobserver variability correlations for InD were strong (R = 0.80, *p* < 0.0001). Conclusions: We provide clinically meaningful reference ranges for InD in subjects with normal LV function, which will emerge as an important screening and assessment imaging tool for a range of HFrEF therapies.

## 1. Introduction

The assessment of left ventricular (LV) ejection fraction (LVEF) using surface echocardiography remains the most common modality of LV systolic function assessment; however, LVEF is a measure of global LV function, and its ability to reproducibly and accurately assess regional LV function remains relatively limited [1]. Global longitudinal strain (GLS) has been incorporated as an adjunct to LVEF to assess overall systolic LV function [2,3,4], and it has been shown to contain information that is integrative to LVEF in patients with heart failure (HF) with reduced ejection fraction (HFrEF) [5,6]. Nevertheless, GLS is a global measure too, and fails to differentiate between the basal, mid- and apical regions of the LV wall. In order to distinguish segmental LV contractility, novel imaging methods are required that enable segment-specific tracking of the motion of the endocardial wall or border. This is particularly important in the HFrEF population, where novel interventions (i.e., left ventriculoplasty devices [7]) target regional LV dysfunction.

Regional myocardial strain analysis via two-dimensional (2D) speckle tracking echocardiography (STE) enables the quantitative evaluation of regional LV function through image-based analysis of myocardial deformation [8,9], facilitating the detection of ventricular dysfunction earlier in the disease process [8]. The STE-derived measurement of longitudinal, circumferential and radial strain has been studied extensively and has led to the determination of normal ranges for different age, sex and disease groups [8]. However, regional strain by 2D STE harbors a high degree of measurement variability in addition to the known intervendor bias [10]. The variability in both 2D and 3D STE regional strain is partly sensitive to the fact that segmental strain is a measure of “differential” local longitudinal displacement (i.e., between the edges of each LV segment), where the inherent acquisition variabilities in STE are amplified.

With these limitations in mind, inward displacement (InD) has been developed as a novel imaging tool [11,12] that is based on absolute measures of regional motion, rather than derived (i.e., calculated) from differences. InD can be evaluated using either STE or feature tracking (FT) in cardiac magnetic resonance (CMR) or computed tomography (CT) imaging and enables a precise measurement of the regional displacement of the LV endocardial border with respect to the true LV centerline [12]. Indeed, InD was expressly designed as an optimal mathematical measure for the evaluation of regional function. Thus far, InD has been investigated in one cohort study and was demonstrated to hold significant promise in the evaluation and planning of left ventriculoplasty therapies for HFrEF patients as well as for the post-procedural assessment of loco-regional treatment efficacy [13]. One clinical case of its application in a patient with myocardial infarction showed a marked reduction in InD in dyskinetic segments [12]. More recently, a systematic clinical study demonstrated the incremental prognostic value of InD in patients with ischemic heart disease and its potential relevance for risk stratification in patients with ischemic disease [11].

Progress in clinical studies aiming to identify regional alterations requires knowledge of the ranges of normal InD values for each of the 17 LV segments to properly place abnormal observations into clinical context. However, normal InD values are not available yet for clinical use. Therefore, this study aimed to fill this gap and establish normal reference limits for LV segment-specific InD in healthy adults based on CMR imaging data.

## 2. Materials and Methods

### 2.1. Study Population

We studied 120 randomly selected subjects from the UK Biobank and the Heart and Vascular Center of Semmelweis University, Budapest, Hungary, with 10 men and 10 women selected from each of the six age deciles from 20 to 80 years. Included subjects were completely asymptomatic, with no known cardiovascular risk factors, free of cardiac conditions upon screening and with a reported LVEF of 55–70%.

### 2.2. CMR Image Acquisition

1.5 T scanners were used for the CMR scanning of all patients of UK Biobank and the Heart and Vascular Center of Semmelweis University. The field of view was adjusted according to body size; slice thickness was 8 mm (mm) without an interslice gap; and pixel size was between 1.5 and 2.0 mm in plane. Repetition Time (TR) and Time to Echo (TE) were set to minimal depending on the gradient system performance and other patient-related characteristics. TR and TE settings were usually set to between 1.4 and 1.6 milliseconds (ms) and 2.7 and 3.2 ms, and temporal resolution exceeded 40 ms.

After assignment to the different age and gender groups, the image quality of Cine SSFP MR long-axis images for 2CH, 3CH and 4CH was assessed for each subject. If image quality was found to be insufficient (e.g., due to a misangulation of slices or visual artifacts hindering the border definition of the LV), the subject’s dataset was replaced with that of another randomly selected subject matched for age, sex and LVEF.

### 2.3. CMR Analysis

**Figure 1:**
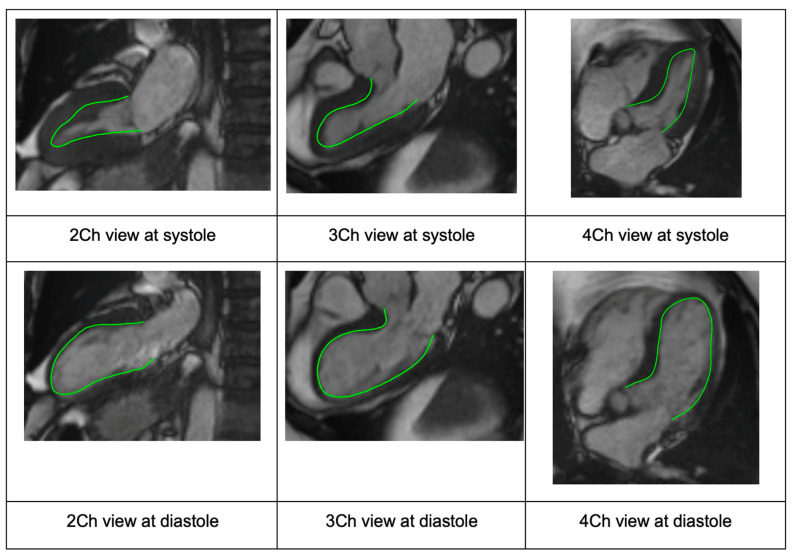
Case example showing the standard long-axis views via CMR with endocardial border detection (green line) used to generate InD segment values (2CH, 3CH and 4CH views from left to right, respectively). The overlapping long-axis views are shown for systole (row 1) and diastole (row 2). Abbreviations: CMR, cardiac magnetic resonance; CH, chamber; InD, inward displacement.

For each subject, the three LAX image series (2CH, 3CH and 4CH) from CMR scans were transferred to an image analysis workstation (Medis Suite 4.0.50.0, QMass 8.1.148.0, Medis Medical Imaging Systems BV, Leiden, The Netherlands). Automated endocardial border LV contour detection was conducted for each of the LAX series for the end-diastolic (ED) and end-systolic (ES) frames (Figure 1). If needed, manual corrections were performed on the automatically detected endocardial contours. The subjects were assigned evenly to three experienced analysts (SM, AT and RL) to perform the manual analysis steps. After contour detection, the InD feature tracking computation was performed using the QStrain application (QStrain MR 4.1.16.0, Medis Medical Imaging BV, Leiden, The Netherlands) [12]. Of note, the Medis InD solution, which is part of the Medis QStrain application, is available for CMR, computed tomography and echocardiography. Based on the endocardial contours, the end-diastolic volume (EDV), end-systolic volume (ESV), LVEF and GLS were derived.

### 2.4. Inward Displacement

**Figure 2:**
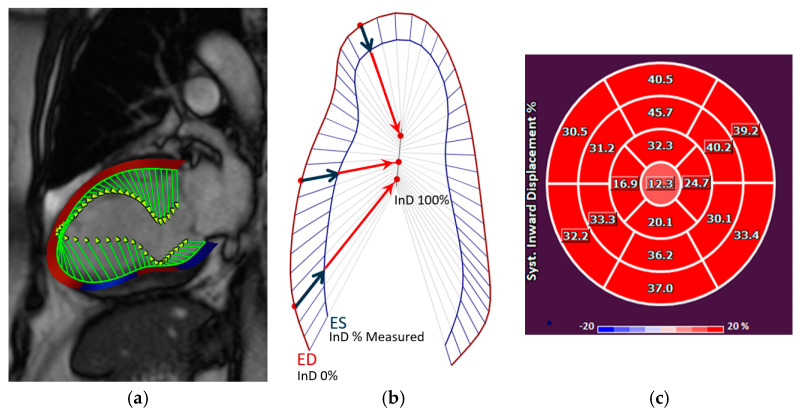
(**a**) The inward motion of the endocardial wall towards the LV “center of contraction” (demonstrated by the green lines) represents the effective result of LV contraction, comprising a combination of longitudinal and radial motion that gives rise to the reduction in the LV volume. InD is evaluated using Medis’ feature-tracking technology. (**b**) InD is a value defined for each point of the endocardial border from end-diastole (ED) to end-systole (ES), given by the component of the displacement vector that is directed toward the LV center. The position of the LV centerline is located along the LV axis with a position that varies from one half to two-thirds of the base-to-apex distance, for the basal to the apical regions, respectively. Regional InD was measured in millimeters (mm) and is expressed as a percentage (%); 0 % represents the absence of contraction and 100% corresponds to a theoretical limit at which the LV shrinks to zero volume at its centerline. InD is measured starting from the end-diastolic (ED) frame. Its value normally increases in each segment during systole to reach a positive peak value at end-systole (ES). It then decreases during the phases of diastole to eventually return to zero at end-diastole. (**c**) After InD is measured for the three standard long-axis views of the LV, the results are plotted on a standard AHA 17-segment bullseye diagram to depict the measured value for each LV segment. Abbreviations: LV, left ventricular; InD, inward displacement; ED, end-diastole; ES, end-systole.

**Figure 3:**
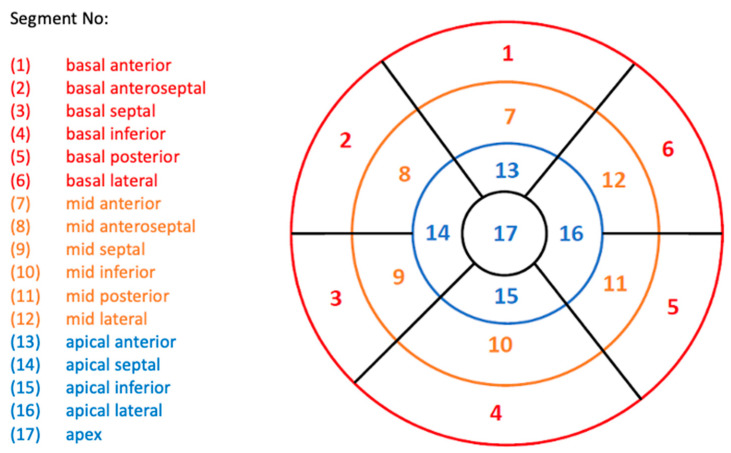
Standard AHA Guidelines bullseye plot used for LV assessment, showing the 17 LV segments. Segments are categorized into three LV regions: basal, red; mid-cavity, orange; and apical, blue. Abbreviations: AHA, American Heart Association; LV, left ventricle.

InD assesses the degree of inward endocardial wall motion from end-diastole until end-systole towards the true LV center of contraction (Figure 2a,b). For each of the standard 17 LV segments of the standard AHA Guideline bullseye plot [14] (Figure 3), InD was measured in mm and is expressed as a percentage (Figure 2b,c). The arithmetic average of InD was obtained for each of the 17 segments. The absence of segmental LV contraction is expressed as 0% (i.e., akinesis), whereas negative percentages imply the dyskinesis of the LV wall. InD of 100% corresponds to a theoretical limit at which the LV shrinks to zero volume.

To study interobserver variability, measurements were repeated for 12 randomly selected subjects by a different analyst. Repeated measurements for all 12 subjects were divided between three analysts in total.

### 2.5. Statistical Analysis

Statistical analysis was performed using SPSS Statistics (IBM SPSS Statistics 26). Continuous data are reported as mean ± standard deviation (SD) or as median (interquartile range (IQR)). Categorical outcomes are expressed as frequencies and percentages. A comparison between male and female healthy subjects was conducted using an unpaired t-test or Mann–Whitney U test for normally distributed and non-normally distributed data, respectively. In all cases, *p* < 0.05 was considered statistically significant. Linear regression and Bland–Altman analyses were conducted in order to investigate any possible relationship of the discrepancies between InD measurements of different observers (i.e., interobserver variability).

## 3. Results

### 3.1. Subject Selection

Of 141 screened CMR scans, 120 were of sufficient quality for InD analysis, which comprised the study population. Of the 120 included subjects, 80 subjects were included from UK Biobank (corresponding to the age deciles from 41 to 80 years) and 40 subjects were included from the Heart and Vascular Center of Semmelweis University (corresponding to the age deciles from 21 to 40 years).

### 3.2. Baseline Characteristics

Table 1 summarizes the demographic data of the studied population. A total of 60 men (mean age 51 ± 17 years) and 60 women (mean age 51 ± 17 years) were included (*p* = 0.89). Men had a larger BSA compared to women (2.0 ± 0.1 vs 1.7 ± 0.2 m^2^, *p* < 0.0001). The LVEF was not significantly different between men and women (66.2 ± 5.9% vs. 67.5 ± 4.9%, respectively, *p* = 0.19). Both the LV end-diastolic and end-systolic volumes in men were greater than those in women (164.4 ± 30.3 vs. 127.9 ± 24.5 mL, *p* <0.0001; 56.0 ± 16.1 vs. 41.9 ± 11.7 mL, *p* <0.0001).

### 3.3. Segmental InD Values

**Figure 4:**
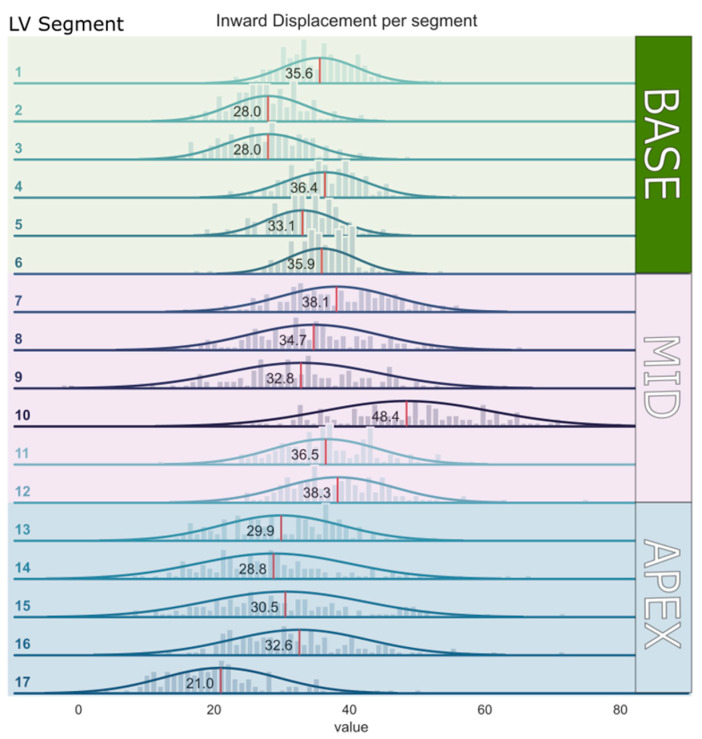
Mean inward displacement values with the corresponding 95% confidence intervals per segment of the left ventricular wall (from segment 1–17).

The mean segmental InD values for each of the 17 LV segments for the entire study population are depicted in Table 2 and ranged from 21.0 ± 8.6% (lowest, apical segment 17) to 48.4 ± 12.4% (highest, mid-cavity segment 10). The mean segmental InD values with the corresponding 95% confidence intervals are shown in Figure 4. The average InD for all segments combined in the entire study population was 33.4 ± 4.3%. InD was found to be higher in the basal and mid-cavity LV segments (32.8 ± 4.1% and 38.1 ± 5.8%, respectively) compared to the apical LV segments (28.6 ± 7.7%) (Table 3). The reference ranges of segmental InD for all age categories are presented in Supplementary Table S1.

### 3.4. Relationship between LV Volumes and InD

Both univariable and multivariable linear regression analyses for volume indices that were adjusted for age and sex showed that a greater average left ventricular end-diastolic volume index (LVEDVI) and left ventricular end-systolic volume index (LVESVI) correlated with significantly lower average InD values (HR: −0.08; 95% CI: −0.13 to −0.02; *p* = 0.007 and HR: −0.46; 95% CI: −0.54 to −0.37; *p* < 0.0001 for multivariable analysis for LVEDVI and LVESVI, respectively).

### 3.5. Interobserver Variability

**Figure 5:**
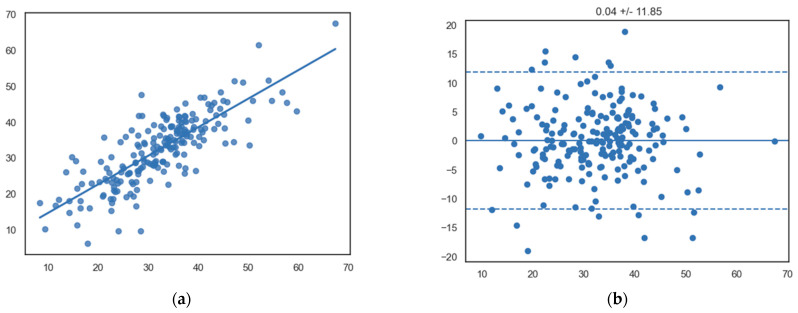
(**a**) Scatterplot showing a significant linear relationship between InD measurements between observers (x-axis, observer 1; y-axis, observer 2) (R = 0.80, *p* < 0.0001). (**b**) Bland–Altman plot showing the mean differences of segmental InD measurements between different observers (x-axis, mean of two measurements; y-axis, difference between two measurements).

Interobserver correlations for InD were strong and significant (R = 0.80, *p* < 0.0001) (Figure 5).

## 4. Discussion

The present study presents a validation of the reference ranges of InD across each of the 17 LV segments in 120 subjects without risk factors or documented heart disease with normal LV function. InD represents a novel method of accurate regional LV assessment. Segment-specific InD values are presented, demonstrating that the majority of LV “pumping” derives from the basal and mid-wall LV segments. The imaging platform and methodology used to calculate InD were highly reproducible with good interobserver variability. These normal segment-specific InD ranges will be a valuable reference tool in the evaluation and quantification of segment-specific (abnormal) LV contractility prior to and following a range of established and emerging HFrEF therapies such as hybrid or minimally invasive left ventricular reconstruction [11,12].

The emergence of a range of therapies that target the HFrEF patient via achieving physical reverse LV remodeling across a specific part of the LV requires an accurate, reproducible regional and segment-specific imaging approach to assess the LV response. The pattern of LV reverse remodeling is heterogenous and can occur remotely (at a distance) following a specific left ventriculoplasty procedure [15]. Currently, there are no established, validated means of assessing regional, segment-specific LV function.

The metrics of regional mechanical function based on strain measured on any imaging modality (CMR, CT or Echo) are highly variable because longitudinal segmental strain is a differential measure of longitudinal motion and torsion between nearby points (e.g., at the edges of a segment), where displacements are dominated by mitral annular motion or global rotation, with differences being highly sensitive to local tracking inaccuracies. In addition, measurements of regional strain when applied to STE imaging are further limited by the various known shortfalls of echocardiographic imaging (e.g., foreshortening and poor acoustic windows) and its reproducibility. Hence, InD was recently developed to measure the regional displacement of the endocardial border towards the LV centerline with respect to the standard three long-axis views [12]. Since the segmental value of InD is an average of absolute inward displacements of all points belonging to the LV segment, its value effectively describes the segmental contribution to contraction not influenced by neighboring segments or basal plane motion and, being an average of displacements rather than a difference, these measurements are less influenced by local tracking errors. The same InD software applied within the scope of this study to CMR imaging can be similarly applied to CT and echocardiography, overcoming the inaccuracies of all currently available strain packages.

So far, InD has only been investigated in one cohort study in which it was applied in a HFrEF population before and after left ventriculoplasty interventions [13] and in patients with ischemic heart disease [11,12], demonstrating its potential relevance in diseases associated with regional dysfunction. The current study describes InD measured on CMR in subjects with normal hearts in order to better place abnormal InD measurements into a clinical context. InD was found to be higher in the basal and mid-cavity LV segments compared to the apical LV segments, which represents the normal contraction of the LV that predominantly originates from the base and mid-LV wall [16,17], whereas the apex show minimal longitudinal or radial displacement in the normal non-operated left ventricle. In line with this, the septal segments are expected to contract the least, which was also demonstrated by our results.

The present study demonstrated that higher average LVEDVI and LVESVI correlated significantly with lower average longitudinal InD values. These findings are in accordance with the observations of our previous InD analysis in a HFrEF cohort, in which we demonstrated that larger LV volumes before LV reconstruction correlate to relatively lower InD values [13]. Similarly, increased InD measurements following left ventriculoplasty correlated well with the observed significant LV volume reductions in the same HF population.

Eventually, InD might represent an integration with STE for assessing segmental LV function since precise endocardial border detection using an automated image-processing system minimizes the shortcomings of TTE. However, although endocardial border LV contour detection is automated for InD, manual corrections can be performed on the automatically detected endocardial contours in the ED and ES phases in the CMR acquisition series. Approximately 10% of the endocardial contours were corrected manually in this study. This can contribute to small differences between different InD measurements performed by various observers. Furthermore, a difference between observers in ED and ES phase selection can partly contribute to interobserver variability. In this study, a good correlation was found between the InD measurements of three different observers, with only small mean differences of segmental InD measurements between observers. Further validation of normal InD values with acquired data from multiple observers across larger sample sizes in the future will undoubtedly fine-tune the current reference ranges that we present. Nevertheless, with the use of these established reference ranges, abnormal segmental InD values can be detected more easily. This facilitates the evaluation and quantification of segment-specific regional wall motion abnormalities and can aid in the planning of a range of left ventriculoplasty therapies for HFrEF patients.

### Limitations

The present analysis is an observational retrospective study in a combined cohort of 120 patients with normal global LV function. As patients in this cohort were selected based on the presence of an adequately functioning LV, a healthy selection bias is present, and therefore the cohort does not fully reflect the “normal” aging population. Although this is strongly indicative of adequate cardiac function in the absence of major cardiovascular problems, the detailed patient profiles of the selected population are unavailable. Nevertheless, the aim of this study was to calculate normal ranges for InD across each LV segment in these subjects, for which additional data were not deemed essential. InD values were derived from CMR images and not from CT scanning. As the image processing of InD is similar in both CMR and CT and depends on adequate contour detection, these reference ranges are thought to be widely applicable across both imaging methods and represent the only currently available InD data in subjects deemed to be normal. As the concept of InD can also be applied to STE imaging, it is expected that similar values might be used for such cases; however, further studies are needed to support this.

## 5. Conclusions

This is the first study that provides applicable reference ranges for InD in the normal left ventricle across all the 17 LV segments. These data will serve as a valuable reference tool for measuring specific LV segmental function. These normal values may allow us to better evaluate impaired segment-specific contractility and will aid in the identification of procedural suitability and evaluation of the effect of a range of HFrEF therapies, including left ventriculoplasty procedures. By overcoming the multiple shortfalls of regional strain analysis, InD may ultimately emerge as the gold standard for regional LV functional assessment.

## Figures and Tables

**Table 1:** Baseline characteristics of the included subjects.

Variable	All Patients (*n* = 120)
Age (y)	50.7 ± 16.9
Male (%)	50%
Patient weight (kg)	72.0 ± 13.3
Patient height (cm)	172.2 ± 9.1
BSA (m^2^)	1.8 ± 0.2
EDV * (mL)	146.1 ± 33.0
ESV * (mL)	48.9 ± 15.7
EF * (%)	66.8 ± 5.4

Values are mean ± standard deviation (SD). Abbreviations: BSA, body surface area; cm, centimeter; EDV, end-diastolic volume; EF, ejection fraction; ESV, end-systolic volume; kg, kilogram; m, meter; mL, milliliter; y, years; *, derived from long-axis view on cardiac magnetic resonance imaging.

**Table 2:** Reference ranges of inward displacement for each of the 17 segments of the left ventricular wall.

Segment	Region	Mean (%)	SD (%)
Segment 1	Basal anterior	35.6	5.6
Segment 2	Basal anteroseptal	28.0	5.7
Segment 3	Basal inferoseptal	28.0	6.4
Segment 4	Basal inferior	36.4	6.1
Segment 5	Basal inferolateral	33.1	5.3
Segment 6	Basal anterolateral	35.9	5.1
Segment 7	Mid-anterior	38.1	8.4
Segment 8	Mid-anteroseptal	34.7	9.7
Segment 9	Mid-inferoseptal	32.8	11.4
Segment 10	Mid-inferior	48.4	12.4
Segment 11	Mid-inferolateral	36.5	7.9
Segment 12	Mid-anterolateral	38.3	8.3
Segment 13	Apical anterior	29.9	8.9
Segment 14	Apical septal	28.8	11.2
Segment 15	Apical inferior	30.5	11.7
Segment 16	Apical lateral	32.6	10.1
Segment 17	Apex	21.0	8.6
Average	All segments	33.4	4.3

Values are mean and standard deviation (SD).

**Table 3:** Reference ranges of average inward displacement for the basal, mid- and apical regions of the left ventricular wall.

Region	Mean (%)	SD (%)
Base	32.8	4.1
Mid	38.1	5.8
Apex	28.6	7.7

Values are mean and standard deviation (SD).

## Data Availability

The data presented in this study are available on request from the corresponding author. The data are not publicly available due to data protection.

## Supplementary Materials

The following supporting information can be downloaded at: https://www.mdpi.com/article/10.3390/jcdd10120474/s1, Figure S1: Scatterplot showing the correlation between inward displacement and ejection fraction; Table S1: Reference ranges of InD for each of the 17 LV-segments for all age categories.
