# Supplementary material for: Reference Values for Inward Displacement in the Normal Left Ventricle: A Novel Method of Regional Left Ventricular Function Assessment
Source: J Cardiovasc Dev Dis. 2023 Nov 24;10(12):474. doi: 10.3390/jcdd10120474 (PMC10744219; doi:10.3390/jcdd10120474)
Supplement: Supplementary file 1 [file jcdd-10-00474-s001.zip › jcdd-2699741-supplementary.pdf]

Figure S1. Scatterplot showing the correlation between inward displacement and ejection fraction.

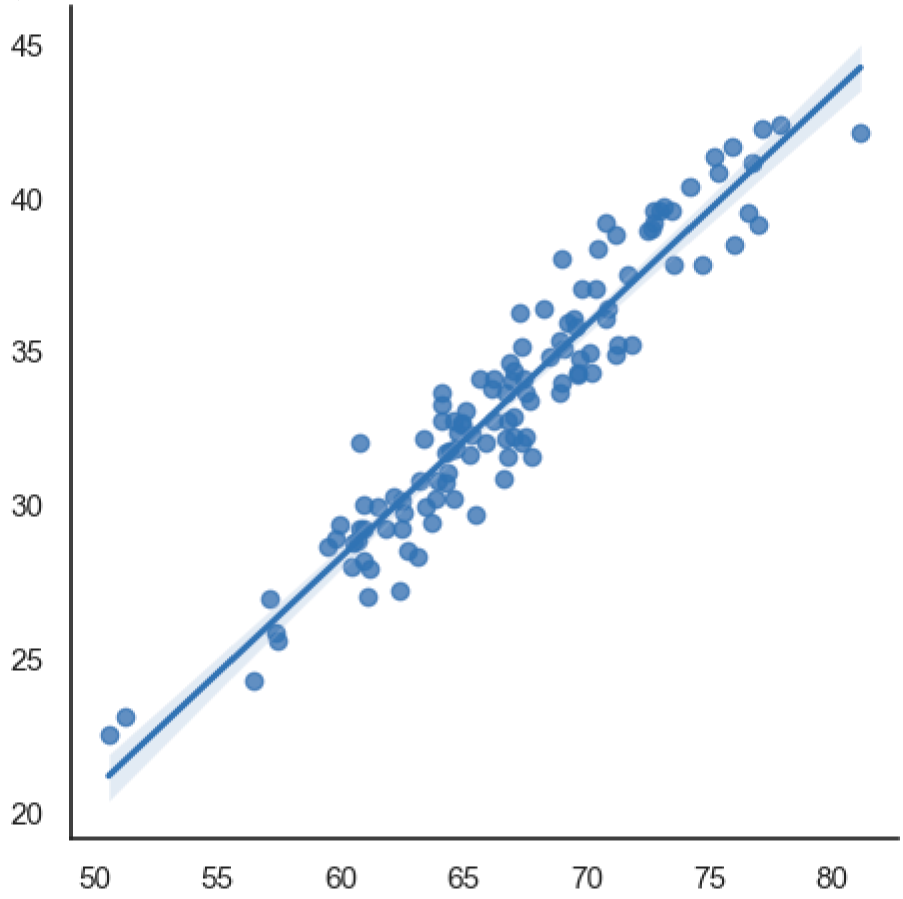

Y-axis, Average Inward Displacement (%). X-axis, Ejection Fraction (%). R = 0.95; p < 0.0001.

Table S1. Reference ranges of InD for each of the 17 LV-segments for all age categories

	Age	21-30 (y)	31-40 (y)	41-50 (y)	51-60 (y)	61-70 (y)	71-80 (y)
Segment 1	mean	35.5	34.9	37.3	35.6	36.8	33.6
	sd	5.2	6.4	3.5	4.5	7.4	5.7
Segment 2	mean	27.3	27.8	29.5	28.2	30.2	24.9
	sd	5.7	5.4	5.1	4.5	7.0	5.6
Segment 3	mean	27.1	27.5	28.6	28.2	30.3	26.2
	sd	5.7	6.2	6.3	6.3	7.0	6.6
Segment 4	mean	37.6	35.0	38.4	36.8	36.9	33.4
	sd	5.9	6.6	6.4	5.3	5.8	5.9
Segment 5	mean	30.8	32.1	35.8	33.7	34.0	31.9
	sd	5.1	7.1	2.8	4.4	5.2	5.5
Segment 6	mean	33.9	35.6	37.6	36.0	37.7	34.7
	sd	6.0	5.4	5.3	3.8	5.2	4.4
Segment 7	mean	34.4	33.4	38.7	38.1	43.7	40.2
	sd	6.5	10.0	8.3	7.1	7.4	7.1
Segment 8	mean	29.7	31.3	34.0	34.7	42.7	35.9
	sd	7.8	10.0	7.4	8.4	11.4	8.0
Segment 9	mean	26.5	25.0	32.2	36.5	37.5	39.4
	sd	9.2	9.7	9.6	8.2	12.6	11.0
Segment 10	mean	43.1	41.8	49.3	52.3	54.3	49.8
	sd	10.9	11.3	14.3	11.1	10.2	12.1
Segment 11	mean	33.7	36.4	38.8	34.6	37.8	37.6
	sd	5.7	7.6	8.6	6.8	9.7	8.3
Segment 12	mean	33.6	36.0	40.2	39.1	39.7	41.0
	sd	4.6	7.7	7.5	6.0	11.7	8.6
Segment 13	mean	26.6	28.3	31.7	28.4	32.5	32.0

	sd	7.9	8.0	9.0	9.9	10.3	7.2
Segment 14	mean	25.7	27.4	28.2	26.9	30.5	34.0
	sd	13.3	8.2	11.1	12.2	10.4	10.6
Segment 15	mean	27.3	26.8	30.9	29.3	34.2	34.6
	sd	7.9	12.6	9.9	11.7	15.0	10.6
Segment 16	mean	29.7	30.5	31.9	28.3	37.1	38.2
	sd	9.5	8.5	8.9	8.4	13.2	8.2
Segment 17	mean	19.0	19.3	20.9	17.7	23.7	25.4
	sd	8.9	6.2	8.7	8.5	9.8	7.7
Average	mean	30.7	31.1	34.4	33.2	36.5	34.9
	sd	3.6	5.1	3.7	3.4	3.4	3.6

Values are mean or standard deviation (sd). Abbreviations: InD, inward displacement; LV, left ventricular; sd, standard deviation; y, years.
